# Authors’ Response to Peer Reviews of “Google Trends as a Predictive Tool for COVID-19 Vaccinations in Italy: Retrospective Infodemiological Analysis”

**DOI:** 10.2196/38695

**Published:** 2022-04-19

**Authors:** Alessandro Rovetta

**Affiliations:** 1 R&C Research Bovezzo Italy

**Keywords:** COVID-19, epidemiology, Google Trends, infodemiology, infoveillance, Italy, public health, SARS-CoV-2, vaccinations, vaccines, social media analysis, social media

*The author’s response to peer-review reports for “Google Trends as a Predictive Tool for COVID-19 Vaccinations in Italy: Retrospective Infodemiological Analysis.”*

## Round 1 Review

### Reviewer M [1]

Comment: The subject of the brief paper [2] “Google Trends as a Predictive Tool for COVID-19 Vaccinations in Italy: a Retrospective Infodemiological Analysis” is timely and valuable to the audience of JMIRx Med. Overall, the paper is well structured, reads exceptionally well, and covers the existing literature quite well. The analysis of the data is interesting and well documented.

The author of the paper has selected keywords used in the Google Search engine, which could reveal an intention to take a vaccine against COVID-19 in Italy and compared this interest with headlines in the second most read newspaper in Italy. The paper has a transparent and replicable procedure to collect data and do statistical tests.

The results show a marked and significant cross-correlation between web queries on vaccine reservations and actual vaccinations against COVID-19 in Italy. On the other hand, the cross-correlation between vaccine-related news and vaccine web searches is low.

Answer: I thank the reviewer for the comprehensive summary and positive comments regarding this paper.

Minor comment 1: I think that the limitations of this study are much broader than those listed in the work. There is a strong vaccine hesitation movement across different European countries, which could at least be mentioned in the work. The authors only noticed news in a newspaper on rare side effects of vaccination. This is what strongly influences, on the one hand, queries entered into a search engine and, on the other hand, a decrease in the number of vaccinations.

Answer 1: Dear Reviewer, I totally agree on the effects of vaccine hesitancy and the impact of mass media on web queries. In this regard, I have opted to introduce new results in the manuscript. Indeed, keywords related to not getting vaccinated and vaccine booking cancellations have been considered. In particular, it was shown that these keywords represented about 4% of the relative search volume (RSV) of the keyword “prenotazione vaccino” (vaccine reservation). Furthermore, the limitations section has been enriched.

Modified section: Introduction: “At present, monitoring of vaccine adherence is epidemiologically essential, especially considering the growing no-vax movement.”

Modified section: Methods: Data Collection: “Following the previous methods, the keywords ‘disdire vaccino + cancellare vaccino + evitare vaccino + non vaccinarsi + green pass falso + comprare green pass’ (revoke vaccine + cancel vaccine + avoid vaccine + do not get vaccinated + fake green pass + buy green pass) were searched to investigate users' web interest in methods of not getting vaccinated. The first keyword searched was ‘disdire vaccine.’ The other terms have been selected by consulting various possible synonyms in the Treccani.it online dictionary and Google Trends related queries.”

Modified section: Results: “The keywords related to the desire not to get vaccinated registered an average RSV of 4% compared to ‘vaccine reservation.’”

Modified section: Discussion: Limitations: “Finally, although well targeted, there are no guarantees that all the keywords relating to the desire not to be vaccinated have been selected. In this regard, given the broad anti-vaccination movement, many users may not have expressed an online interest in not getting vaccinated.”

### Reviewer O [3]

Comment: The paper uses Google Trends (GT) to identify correlations between search queries and vaccinations. GT has been used previously by others for similar and other problems. The paper is well written. The Methods section can be improved. The Results section has a good explanation.

Answer: Dear Reviewer, thank you for your critical and positive evaluation of this paper.

Comment 1: The novelty of the paper is limited.

Answer 1: Dear Reviewer, I agree that some of the findings in this paper are intuitive. However, I believe that, as scientists, any analysis should not be prejudiced. For this reason, I found it helpful to provide more concrete evidence regarding the possible use of GT as a predictive tool for vaccinations. In particular, in some cases, GT’s reliability has been compromised by spurious correlations with the media hype of related news. This paper provides evidence that well-targeted keywords can overcome such a problem.

Comment 2: The Introduction is short and can be extended to include more relevant studies.

Answer 2: Dear Reviewer, I agree and thank you for this criticism. I have enriched the introduction, trying to provide a thorough background on the topic. If further changes are required, I will be available to integrate them. However, I would like to try not lengthening this section too much to avoid violating the “short paper” structure (which I believe can be communicatively advantageous).

Comment 3: The Methods section needs more details. For instance, how GT works, especially when keywords are two words “vaccine reservation.” Does it search for all queries that include both words vaccine and reservation or vaccine OR reservation, or does it search for an exact match (“vaccine reservation”)? More search terms can be included, such as synonyms of reservation like an appointment or booking. Additionally, how was data normalized? What is lag week?

Answer 3: Dear Reviewer, I thank you very much for highlighting these fundamental issues. I propose the list of strategies I have adopted to solve these problems below.

- Queries: I have provided the URL of the search on GT to facilitate the reproducibility of the analysis. Additionally, I confirm that the Vaccine Reservation and “Vaccine Reservation” queries return highly similar results (proof [4]).
  Modified section: Methods: Data collection: “The final exact queries searched on Google Trends are reported as references.”
- Queries synonyms: The synonyms have been searched on the Treccani.it online dictionary. However, the queries had a much lower RSV (proof [5]). Furthermore, even adding these queries with the “+” operator, the trends remained extremely similar (proof [6]). Since the combination of queries makes it more likely that anomalies will appear in the data sets, I have opted for a single query.
  Modified section: Methods: Data Collection: “Synonyms of the word ‘prenotazione’ (reservation) have been searched on the Treccani.it online dictionary. However, the synonyms queries had a much lower RSV. Besides, even adding them to the original keyword through the ‘+’ operator, the trends remained highly similar. Since the combination of queries makes it more likely that anomalies will appear in the datasets, a single query was chosen.”
- Data normalization: All data sets were normalized to 100 by multiplying individual values by the constant “100/data set maximum value.”
  Modified section: Methods: Statistical Analysis: “All datasets were normalized to 100 by multiplying individual values by the constant ‘100/dataset maximum value.’”
- Lag week definition: The “lag week” was defined as the number of weeks by which a time series was shifted to obtain the maximum correlation with another time series. By doing so, it was possible to estimate the predictive power of one time series over another and the latency between the measurement of the first and the appearance of the second.
  Modified section: Methods: Statistical Analysis: “The ‘Lag week’ was defined as the number of weeks by which a time series was shifted to obtain the maximum correlation with another time series. By doing so, it was possible to estimate the predictive power of one time series over another and the latency between them.”

### Reviewer BL [7]

Comment: This brief paper examines the effective approach to investigating vaccine adherence against COVID-19 via GT. The topic is interesting and important to provide actionable data to the World Health Organization or other related health organizations to prioritize their risk communication efforts. The manuscript is nicely written and easy to understand. These data are of potential interest, but there are some concerns.

Answer: Dear Reviewer, I greatly appreciate the positive feedback and constructive criticism leveled at my paper.

Comments 1, 2, and 3:

1. The methodological strength is poor. It should discuss the overarching sampling method, measures, and procedures to justify the Google and news media content in this study.

2. In line with the methodology concern, the chosen keywords are questionable too.

3. Additionally, there is no rationale for sampling the historical archive of the newspaper “La Repubblica.” Is this the second most read Italian newspaper online?

Answer 1, 2, and 3: Dear reviewer, I sincerely thank you for pointing out these essential points. In this regard, I have made numerous changes and clarifications in the manuscript. I have merged the answers since they are strongly correlated. In particular, thanks also to the previous reviewers' comments, I specified that all the keyword synonyms—found on the Treccani.it online dictionary—were searched on GT and showed very low RSVs compared to the final keyword chosen (proof [5]). The related queries were also consulted for this purpose. Now, I have also specified that “La Repubblica” has been selected as it was the second most read newspaper and, at the same time, the one that provides the most detailed news database. Furthermore, the choice of a single newspaper was based on the fact that previous articles found broad similarities between the news trends of the primary Italian mass media. Indeed, this is compatible with the theory of news competition and increasing returns-to-scale. The keyword used for the search on La Repubblica was chosen since it includes the generic and technical names of the vaccines administered in Italy in the investigated period.

Modified section: Methods: Data Collection: “Synonyms of the word ‘prenotazione’ (reservation) have been searched on the Treccani.it online dictionary. However, the synonyms queries had a much lower RSV. Besides, even adding them to the original keyword through the ‘+’ operator, the trends remained highly similar. Since the combination of queries makes it more likely that anomalies will appear in the datasets, a single query was chosen. [...] In particular, this query includes the generic and proper names of the COVID-19 vaccines administered in Italy during the investigated period.”

Modified section: Methods: Data Collection: “This newspaper was chosen since it represents the second most widely read newspaper in Italy and provides the most detailed news database online. Furthermore, a previous publication showed very similar news trends across primary Italian mass media during COVID-19. Such a result aligns with the theory of news competition and increasing returns-to-scale, which prompts profit-motivated media to publish on hot topics (as of interest to a broad audience). For these reasons, the author of this paper considered the source ‘La Repubblica’ sufficient to represent the Italian media clamor about vaccines.”

Comments 4 and 5:

4. Confounding is a statistical concept that is important to all researchers. The concept of confounding is explained with the help of an amusing but true example. The methods to deal with confounding should be more detailed, with more applications and disadvantages to be examined.

5. The role of the mass media was considered as a confounding factor. Actually, confounding is said to exist when a third factor, known as the confounding variable, explains the association between two variables. One of the results indicated that vaccine reservation queries (VRQs) and news about COVID-19 vaccines have been low and characterized by lags. I am afraid this could be a failure to identify and control for confounding, which could result in the faulty interpretation of study outcomes. So, you really can’t say for sure whether the lack of news influence (ie, from one specific website only) leads to the unwillingness of vaccination.

Answers 4 and 5: Dear Reviewer, I agree both with the importance of clarifying the concept of confounding and that this paper has not been able to analyze all the possible confounders. In this regard, I have substantially modified the manuscript to clarify the role of this research. In addition, to improve the quality of the evidence, I introduced Holm-Bonferroni correction and multiregression analysis. In particular, I kindly invite you to read the modified and new sections, which should be exhaustive from this point of view.

Modified section: Methods: Data Collection: “Following the previous methods, the keywords ‘disdire vaccino + cancellare vaccino + evitare vaccino + non vaccinarsi + green pass falso + comprare green pass’ (revoke vaccine + cancel vaccine + avoid vaccine + do not get vaccinated + fake green pass + buy green pass) were searched to investigate users' web interest in methods of not getting vaccinated. The first keyword searched was ‘disdire vaccine.’ The other terms have been selected by consulting various possible synonyms in the Treccani.it online dictionary and Google Trends related queries.”

Modified section: Methods: Statistical Analysis. “Finally, a multiple regression was used to build the function Y=f(VRH, VRQ) to evaluate the impact of VRH and VRQ on V. Standard errors for the regression coefficients are reported after ‘±.’ Based on previous literature, any causal correlations between the media clamor and web searches should be sought within a maximum of ±3 weeks (acceptability range) . Indeed, the web interest in a topic must arise around the media hype peak to be considered a direct consequence of the latter. Regarding the pairs (VRH, V) and (VRQ, V), the lag acceptability range was fixed at 0 – 8 weeks since it can take up to two months from vaccine booking to administration. Fisher r-to-z transformation (z) was used to compare Spearman coefficients. Since the search for cross-correlations is highly exploratory, the Holm-Bonferroni correction was adopted (m=50 hypotheses). The original *P* values have been reported alongside the adjusted ones (P*) – when P*>.001 – to allow the reader to interpret the data independently.”

New section: Methods: Mass Media Clamor as a Confounding Factor: “As discussed above, there is solid evidence that mass media can significantly impact users' web interests. This fact increases the probability of spurious correlations due to a so-called confounding factor, defined as a ‘hidden’ variable (or set of variables) capable of distorting the true relationship between other apparently (un)correlated variables. In this specific case, media hype can create highly confounding scenarios. For example, a COVID-19 outbreak can generate intense news fanfare, immediately followed by a growing users' web interest in the disease. After seven days, an increase in COVID-19 cases is registered. Examining the sole couple (user interest, COVID-19 cases), it could seem that online searches predicted the increase in infections. However, by introducing the ‘media hype’ variable, it is observed that users' web interest is much more correlated with the latter than with COVID-19 cases. For this reason, media coverage is introduced in this analysis as a possible confounding factor capable of distorting the relationship between V and VRQ. In this regard, it is fair to admit that other confounding factors not considered in this paper could alter such a relationship in complex ways. Nonetheless, at present, to the best of the author's knowledge, media influence is the only widely reported confounding factor in the literature regarding Google Trends. Furthermore, the main research hypothesis is well-targeted, thus reducing the likelihood of spurious correlations.”

Modified section: Results: “The keywords related to the desire not to get vaccinated registered an average RSV of 4% compared to ‘vaccine reservation.’”

Modified section: Discussion: Limitations: “Finally, although well targeted, there are no guarantees that all the keywords relating to the desire not to be vaccinated have been selected. In this regard, given the broad anti-vaccination movement, many users may not have expressed an online interest in not getting vaccinated.”

Other changes: Old results have been modified, and new results have been added.

Comment 6: Another study outcome linked the VRQs and vaccinated for their positive linear relation. Instead of a valuable research question, it sounds like common sense that most laymen would agree with.

Answer 6: Dear Reviewer, I agree that the primary hypothesis is very intuitive. However, my thought is that scientists should not be limited by their own prejudices and that, when possible, even reasonable assumptions deserve to have supporting evidence. For this reason, I thought of writing this short paper to give further strength to such a hypothesis to be able to build more effective infoveillance systems in the future.

Comment 7: Following the abovementioned concern, it is not sustainable that the conclusion shows that GT is a surveillance and prediction tool for vaccine adherence against COVID-19 in Italy.

Answer 7: Dear Reviewer, I modified the conclusion by explicitly writing that the paper provides preliminary evidence. Additionally, I recommend using GT only as a complementary tool.

Modified section: Discussion: Conclusion: “This research provides preliminary evidence in favor of using Google Trends as a surveillance and prediction tool for vaccine adherence against COVID-19 in Italy. Further research is needed to establish appropriate use and limits of Google Trends for vaccination tracking.”

Comment 8: Please list the ethics issue for this study if approved.

Answer 8: Thank you very much for this suggestion.

New section: Ethical Declaration: “This study does not involve human subjects and/or animals. All Google Trends data is anonymized. Therefore, the research does not require approval from a committee. No funding was received. The author declares that he has no conflicts of interest.”

Comment 9: The first letters of a term should correspond to the initials, for example, “vaccine reservation query” (VRQ).

Answer 9: Thank you for having noticed it. I changed to “vaccine reservation query.”

## Abbreviations

GT: Google Trends
RSV: relative search volume
VRQ: vaccination reservation query
